# Supplementary material for: Content-rich biological network constructed by mining PubMed abstracts
Source: BMC Bioinformatics. 2004 Oct 8;5:147. doi: 10.1186/1471-2105-5-147 (PMC528731; doi:10.1186/1471-2105-5-147)
Supplement: Additional File 2 — The original results of the above study (non-essential files are deleted to keep the file size under the limit set by BMC bioinformatics). [file 1471-2105-5-147-S2.bz2 › chilibotAdditionalFile2/dip05/537ID10611280E10886/html/AR_NR2C2.html]

 


 **AR** and **NR2C2** 
  
Found 11 abstracts in PubMed, retrieved 05.  
 

 What does Google say? 
 PDF only 
| .edu only 

---

**Interactive relationship** (e.g. stimulation, inhibition, etc)

**Neutral relationship**- Our aim was to pharmacologically characterize the effect of a nonpeptide NK1 receptor antagonist TAK  [ **NR2C2** ]  637  **aR** , 9R 7 bis trifluoromethyl benzyl, 1 1 tetrahydro 9 methyl 5 4 methylphenyl 7H diazocino g naphthyridine 3 dione and determine key mechanisms of TAK  [ **NR2C2** ]  637 action in the gastrointestinal tract.  Ref: 11861814 J Pharmacol Exp Ther, 2002

**Non-interactive relationship** (e.g. studied together, co-existance, homology, etc.)

- In this study, we investigated the role of tachykinin NK1 receptors in the visceral pain response abdominal muscle contraction caused by colorectal distention in rabbits previously subjected to colonic irritation, using the selective tachykinin NK1 receptor antagonists TAK  [ **NR2C2** ]  637  **aR** , 9R 7 Bis trifluoromethyl benzyl, 1 1 tetrahydro 9 methyl 5 4 methylphenyl 7H diazocino g naphthyridine 3 dione and CP 9 94 2S, 3S 3 2 methoxybenzylamino 2 phenylpiperidine.  Ref: 11861799 J Pharmacol Exp Ther, 2002
- The suppression of TR4  [ **NR2C2** ]  mediated transactivation is selective because TRA16 shows only a slight influence on the transactivation of androgen receptor  [ **AR** ] , glucocorticoid receptor, and progesterone receptor.  Ref: 12486131 J Biol Chem, 2003
- The effect of TAK  [ **NR2C2** ]  637  **aR** , 9R 7 bis trifluoromethyl benzyl, 1 1 tetrahydro 9 methyl 5 4 methylphenyl 7H diazocino g naphthyridine 3 dione, a tachykinin NK1 receptor antagonist, on lower urinary tract function was investigated in cats.  Ref: 11459118 Jpn J Pharmacol, 2001
- Furthermore, by using a chimeric receptor beta 2  **AR**  Rfz 2 containing the ligand binding and transmembrane segments from the beta 2 adrenergic receptor beta 2  **AR**  and the cytoplasmic domains from rat Frizzled 2 Rfz 2, stimulation with the beta adrenergic agonist isoproterenol activates activities of endogenous CaMKII, TAK1  [ **NR2C2** ] , and NLK and inhibits beta catenin induced transcriptional activation.  Ref: 12482967 Mol Cell Biol, 2003
